# Structure and application of antifreeze proteins from Antarctic bacteria

**DOI:** 10.1186/s12934-017-0737-2

**Published:** 2017-08-07

**Authors:** Patricio A. Muñoz, Sebastián L. Márquez, Fernando D. González-Nilo, Valeria Márquez-Miranda, Jenny M. Blamey

**Affiliations:** 1 Fundación Científica y Cultural Biociencia, José Domingo Cañas, 2280, Ñuñoa, Santiago, Chile; 2 Facultad de Química y Biología, Universidad de Santiago de Chile, Alameda 3363, Estación Central, Santiago, Chile; 3 Center for Bioinformatics and Integrative Biology (CBIB), Facultad de Ciencias Biológicas, Universidad Andres Bello, Avenida Republica 239, Santiago, Chile

**Keywords:** Antifreeze proteins, Antarctica, Psychrophiles, Frozen food, Ice binding proteins

## Abstract

**Background:**

Antifreeze proteins (AFPs) production is a survival strategy of psychrophiles in ice. These proteins have potential in frozen food industry avoiding the damage in the structure of animal or vegetal foods. Moreover, there is not much information regarding the interaction of Antarctic bacterial AFPs with ice, and new determinations are needed to understand the behaviour of these proteins at the water/ice interface.

**Results:**

Different Antarctic places were screened for antifreeze activity and microorganisms were selected for the presence of thermal hysteresis in their crude extracts. Isolates GU1.7.1, GU3.1.1, and AFP5.1 showed higher thermal hysteresis and were characterized using a polyphasic approach. Studies using cucumber and zucchini samples showed cellular protection when samples were treated with partially purified AFPs or a commercial AFP as was determined using toluidine blue O and neutral red staining. Additionally, genome analysis of these isolates revealed the presence of genes that encode for putative AFPs. Deduced amino acids sequences from GU3.1.1 (gu3A and gu3B) and AFP5.1 (afp5A) showed high similarity to reported AFPs which crystal structures are solved, allowing then generating homology models. Modelled proteins showed a triangular prism form similar to β-helix AFPs with a linear distribution of threonine residues at one side of the prism that could correspond to the putative ice binding side. The statistically best models were used to build a protein-water system. Molecular dynamics simulations were then performed to compare the antifreezing behaviour of these AFPs at the ice/water interface. Docking and molecular dynamics simulations revealed that gu3B could have the most efficient antifreezing behavior, but gu3A could have a higher affinity for ice.

**Conclusions:**

AFPs from Antarctic microorganisms GU1.7.1, GU3.1.1 and AFP5.1 protect cellular structures of frozen food showing a potential for frozen food industry. Modeled proteins possess a β-helix structure, and molecular docking analysis revealed the AFP gu3B could be the most efficient AFPs in order to avoid the formation of ice crystals, even when gu3A has a higher affinity for ice. By determining the interaction of AFPs at the ice/water interface, it will be possible to understand the process of adaptation of psychrophilic bacteria to Antarctic ice.

**Electronic supplementary material:**

The online version of this article (doi:10.1186/s12934-017-0737-2) contains supplementary material, which is available to authorized users.

## Background

Antarctica is an extreme continent with the coldest temperatures, low precipitations, dryness, and almost completely covered by ice. In this continent, microorganisms dominate the genetic pool and biomass playing an important role maintaining the operation of the ecosystem. Although different microorganisms have been isolated from several places, biological records are available for only a miniscule fraction of them from land and surrounding waters [1].

Psychrophilic microorganisms dominate the majority of Antarctic habitats [2]. For their survival, they have developed several growing strategies on ice including the production of compatible solutes, exopolysaccharides, and antifreeze proteins [3].

Initially, antifreeze proteins (AFPs) were described in marine fishes [4, 5] and defined as cryoprotectants [6]. Currently, AFPs have been obtained from different sources including snow molds fungi [7], sea ice diatoms [8], snow alga [9] and bacteria [10–13].

AFPs are a diverse group of ice-binding proteins that prevent ice growing through the depression of the freezing point of a solution to below the melting point [14]. This difference between freezing and melting point is referred as thermal hysteresis (TH) and results from the adsorption of the AFP on the crystal surface of ice. This interaction causes the ice growth to take place in a convex surface between adjacent AFPs, thus decreasing the freezing point [15]. Additionally, these proteins act at low concentration and they are 200–300 times more effective than ideal solutes [16], being AFPs from insects 10–100 more than those from fish [17].

The full biotechnological potential of AFPs has not been fully exploited [18]. They possess potential applications in cryopreservation [19], as additive in CO_2_ hydrate slurry production [20] and frozen food preparation [21]. On this last case, cucumbers and zucchinis, which have high water content, loose their crispness when they are freeze–thaw cycles, resulting in poor product quality. Antarctica possesses a wide unexplored diversity of microorganisms to search for AFPs in order to produce these proteins in large commercial scales.

This study was focused on searching bacterial AFPs from different places in Antarctica, including continental and insular sites. Several microorganisms were isolated and subjected to continued cycles of freezing and thawing in order to select those who produce AFPs. Crude extracts were prepared from several isolates and TH was determined using a Nanoliter Osmometer. AFP-producing isolates (AFP5.1, GU1.7.1 and GU3.1.1) were characterized using a polyphasic approach and AFPs were partially purified from these microorganisms in order to study their protective effect on cucumber and zucchini samples when they were frozen and thawed in the presence or absence of these proteins. AFP-coding genes were obtained from AFP5.1 and GU3.1.1 allowing deducing amino acids sequences in order to obtain homology models for these proteins and analyze in silico their interaction with ice crystal.

## Methods

### Sampling

Sample collection was performed at different places in insular and continental Antarctica during Chilean Antarctic Scientific Expeditions ECA 50–52. Samples consisting of sediment or small stones were collected aseptically using metallic spoons, and Whirl–Pak bags (8 × 8 inch). Ice samples were taken using a metallic screw and 50 mL plastic tubes. Temperature and pH of the selected area for sampling were measured and registered. Samples were kept at 0–4 °C in a cooler and immediately transported to operation stations for processing.

### Enrichment of environmental samples and bacterial isolation

Solid environmental samples were directly inoculated into 20 mL of TGY medium, containing (g/L) 5.0 tryptone, 3.0 yeast extract, and 1.0 glucose. The pH was adjusted to 7.0. Meanwhile, ice samples were thawed in seawater at 4 °C in order to avoid hypotonic shocking of bacteria, centrifuged at 9000×*g* during 10 min at 4 °C, and inoculated on TGY medium. All cultures were incubated aerobically at 4 °C for 4 weeks or until microbial growth were observed.

Enriched cultures were isolated using serial dilutions on liquid TGY and streaking on plates of TGY supplemented with 1.5% (p/p) of agar. Colonies were transferred to a liquid TGY medium. Incubations were performed at 4 °C. These procedures were repeated until a single and homogeneous morphology was observed.

### Freeze–thaw resistant bacteria selection

Isolates were exposed to repetitive freeze–thaw cycles between −20 and 4 °C in order to select which microorganisms have cryoprotective abilities, including AFPs production, according to the protocol described by Wilson et al. [22].

### Microbial identification and characterization

Genomic DNA from the isolates was extracted by the chloroform:isoamyl alcohol method [23]. 16S rRNA gene was amplified by PCR using bacteria specific primers 27F and 1492R [24]. The reaction mix contained 2.5 U Taq DNA polymerase, 200 μM of each deoxynucleotide (dATP, dCTP, dGTP and dTTP), 1× reaction buffer, 0.75 mM MgCl_2_ and 0.5 mM of each primer. PCR consisted of 45 cycles: 95 °C for 45 s, 55 °C for 45 s and 72 °C for 45 s. A final elongation step of 72 °C for 10 min was included. Amplification reactions were carried out using a Palm Gradient Cycler (Corbett). PCR product was observed on 1.0% agarose gel prepared in 1× TAE buffer (40 mM Tris–acetate, 10 mM EDTA) and visualized under UV light using a 1× GelRed (Biotium) in TAE buffer. PCR products were sequenced using the primers described above and manually edited using ChromasPro software (Technelysium Pty Ltd.) for final sequences of 1200 bp. Partial sequences were compared to GenBank using Blastn software.

A phenotypic characterization was performed according to the procedure described by Correa-Llantén et al. [25].

### TH measurement

TH was measured using a Nanolitre Osmometer (Otago Osmometers) using the procedure described by Braslavsky and Drori [26].

### AFP induction and purification

The isolates GU1.7.1, GU3.1.1 and AFP5.1 were grown in 10 L of TGY medium without shaking. The cultures were incubated at 15 °C during 2 weeks in order to increase the cell density. After, bacteria were kept at 2–4 °C to induce the production of AFPs. Cells were centrifuged at 9000×*g* during 10 min at 4 °C. They were suspended in buffer A (50 mM Tris–HCl pH 8.0 and 10% glycerol) containing lysozyme (1 mg/mL) and DNase I (10 μg/mL) and were incubated at 37 °C for 1 h. Samples were sonicated (Branson Sonifier 450) on ice during 10 min, and cell debris were removed by centrifugation at 9000×*g* for 20 min. Crude extract was loaded onto a column (Pharmacia, C 16/20) of Q-Sepharose Fast Flow (Pharmacia Biotech) equilibrated with buffer A. AFPs were eluted using a linear gradient (90 ml) of 0–1 M NaCl in buffer A. AFP started to elute as 0.67 and 0.77 M NaCl was applied to the column for AFP5.1 and GU1.7.1, respectively. TH was measured as is described above. Two fractions that showed TH were eluted when 0.54 and 0.70 M NaCl were applied to the column loaded with GU3.1.1 crude extract. Protein concentration was determined using the method described by Bradford [27].

### Molecular mass determination

Peaks from the Q-Sepharose Fast Flow column containing AFPs were concentrated to a final volume of 0.5 mL by ultrafiltration using a10-kDa Amicon cellulose filter (Millipore), and applied to a column (GE Healthcare, Tricorn 10/600) of Superdex-200 (Pharmacia Biotech) equilibrated with buffer A containing 0.2 M NaCl. The column was calibrated using lysozyme (14.3 kDa), egg ovalbumin (45.0 kDa), bovine serum albumin (66.0 kDa), bovine glutamate dehydrogenase (300.0 kDa) and urease (545.0 kDa) as the standard proteins. TH and protein concentration were measured in each fraction as is described above.

### Effect of AFPs on frozen zucchini and cucumber fruits

Sample pieces (approximately 5 × 5 × 0.5 mm) were cut from zucchini and cucumber fruits with a scalpel and rinsed in deionized water in order to remove cell debris and cellular content released by the mechanical damage of sectioning. Samples were incubated in the presence of 0.1 mg/mL of AFPs from AFP5.1, GU1.7.1, GU3.1.1, a commercial type-III AFP (A/F Protein Inc.) or buffer A at −20 °C during 16 h. Samples were thawed at 4 °C for 4 h, washed in buffer A and stained with toluidine blue O (TBO) or neutral red (NR) according to procedures described by González et al. [28] to determine the effect of AFPs over plant cell integrity and cell viability (CV), respectively.

Integrity of cell walls was visualized using TBO staining. Samples were mounted to microscope slides with a small drop of deionized water and a drop of 0.025% TBO in 1% of Na_2_B_4_O_7_·10H_2_O during 1 min and then rinsed with deionized water. Samples were covered with a cover slip. Excess of water was removed with tissue paper. Micrographs were observed immediately in a light microscope at 4× magnification.

NR is a lipophilic dye that is uncharged and unprotonated in alkaline solutions. It diffuses across cell membranes and accumulates in vacuoles, where the acidic pH protonates the dye, appearing a dark red color in vacuoles in living cells that can maintain pH gradients [28]. Samples were transferred to a solution of 0.04% NR in 0.2 M mannitol–0.01 HEPES (*N*-[2-hydroxyethyl]piperazine-*N*-[2-ethane-sulfonic acid]) buffer pH 7.8 during 2 h. Samples were washed with 0.2 M mannitol–0.01 HEPES buffer, transferred to microscope slides with a small drop of water and covered with a coverslip and observed immediately in a light microscope at 4× magnification. For CV, 15 micrographs were used in order to count living (red to pink cells) and not-viable cells (not stained cells). Approximately, 70–90 cells and 110–130 cells were counted for cucumber and zucchini, respectively, in each micrograph. CV is represented as the percentage of red-stained living cells over the total number of cells counted.

### Whole genome sequencing and annotation

Genomic DNA from the three isolates was extracted as described above and further sequenced at Georgia Genomics Facility (Georgia, USA) using Illumina Miseq:PE300 technology (paired-end reads, 300 bp) using DNA Nextera XT libraries.

Prior to assembly, all reads were trimmed in order to remove any low quality bases from 3′-end using the trimming tool TrimGalore (http://www.bioinformatics.babraham.ac.uk/projects/trim_galore/). Only reads with a quality score Q >28 were considered for the final assembly. Reads were assembled de novo using Velvet assembler [29]. No reference genomes were used. Assembled genomes were further annotated using the automatic annotation server RAST [30].

### AFP sequences identification

For the identification of putative AFP sequences, a local BLAST database was built from a set of 850 protein sequences found in Uniprot database matching the following keywords: antifreeze protein, ice-binding protein and ice-structuring protein. Open reading frames from the sequenced genomes were predicted using PRODIGAL software [31], translated and blasted against the local database built using Blastp [32]. NCBI’s Conserved Domains Database (CDD) [33] was used for the identification of conserved domains in sequences. T-Coffee server [34] was used for multiple sequence alignments in combination with PROMALS [35] for secondary structure prediction of the sequences.

### Homology modeling

Amino acid sequences of three putative AFPs found in GU3.1.1 and AFP5.1 genomes were used as targets for homology modeling using the SWISS-MODEL homology modeling web server [36], because of their structural similarity to other known AFPs which structural data are available. The best templates for each protein were selected according to the Global Model Quality Estimation (GMQE) score estimated by the server before the models were built. The 3D models generated were structurally optimized by means of energy minimization using NAMD molecular dynamics software [37] and further checked for stereochemical quality by Ramachandran plot analysis using RAMPAGE server [38].

### Docking and molecular dynamics simulations

Molecular models of AFPs/hexagonal ice crystals were built using the atomic coordinates from the optimized structures of homology models initially obtained and the coordinates for ice/water from the freely available model proposed by Kuiper et al. [39]. Ligands and grid box for docking were prepared using AutoDock Tools [40] scripts through PyMOL plugin. Polar hydrogen atoms and Gasteiger charges were added to the proteins and all possible torsion were set as inactive (rigid docking). Docking simulations were performed using AutoDock Vina 1.1.2 [41]. Molecular dynamics simulations were carried out by means of NAMD 2.11 [37] using CHARM27 force field [42] and TIP4P water model. Each system was minimized until reach a convergence and molecular dynamics runs were carried out for 150 ns under a NPT ensemble. Langevin thermostat and Langevin piston [43] were used to maintain a constant temperature of 225 K and a constant pressure of 1 atm. The equations of motion were integrated with a 2.0 fs time step along with SETTLE [44] and RATTLE algorithms [45] to constrain the geometry of the water molecules and the length of covalent bonds to hydrogen atoms. Non-bonded energies were calculated using particle-mesh Ewald full electrostatics [46] (grid spacing <0.13 nm) and a smooth (0.7–0.8 nm) cutoff of the Lennard-Jones energy. Long-range electrostatic interactions were calculated every three steps using a multiple-time stepping scheme [37].

Radial distribution function (RDF) g(r), implemented in VMD, specifically, the g_OO_(r) distribution, representing the oxygen–oxygen atomic distances among water molecules, were used to describe the water state in the solvation shells surrounding the AFPs.

## Results

### Isolation and characterization of AFPs-producing bacteria

103 isolates were obtained from different places in insular and continental Antarctica during Chilean Antarctic Scientific Expeditions ECA 50–52, including Union Glacier, King George Island, Greenwich Island, Deception Island and Livingstone Island. Repetitive freezing–thawing cycles were employed to select cryoprotectants-producing bacteria, obtaining 17 isolates able to survive to this selection procedure (Table 1). Small TH values (0.04–0.09) were registered from crude extracts of microorganisms that were unable to survive the selection procedure. From this group of bacteria, eight showed TH of 0.3–0.5 °C. The highest TH was measured in crude extracts from GU1.7.1, GU3.1.1 and AFP5.1. These last microorganisms were selected for further studies.

**Table 1:** Genus identification of freeze–thaw resistant bacteria and TH measured from their crude extracts

Isolate	Genus identification using Blastn	TH (°C)
AFP2.1	*Pseudomonas*	0.35
AFP5.1	*Pseudomonas*	0.41
AFP7.1	Uncultured bacteria	0.22
AFP8.3	*Pseudomonas*	0.13
ID4.3	*Pseudomonas*	0.36
PH14.1	*Pseudomonas*	0.12
PA32.3	*Pseudomonas*	0.15
BR40.1	Uncultured bacteria	0.30
GU1.2.1	*Arthrobacter*	0.17
GU1.6.1	*Sporosarcina*	0.14
GU1.7.1	*Sphingomonas*	0.50
GU3.1.1	*Plantibacter*	0.46
GU3.1.2	*Arthrobacter*	0.11
GU3.1.3	*Arthrobacter*	0.17
GU3.2.1	*Plantibacter*	0.38
GU3.2.2	*Plantibacter*	0.31

TH was determined using a Nanolitre Osmometer

A polyphasic approach (Table 2) was used in order to identify and characterize these microorganisms. GU3.1.1 rods were positive to Gram staining; meanwhile GU1.7.1 and AFP5.1 were Gram-negative rods. Bacteria were identified by PCR using specific primers for Bacteria domain, as members of genera *Sphingomonas*, *Plantibacter* and *Pseudomonas*, respectively. Genus affiliation was confirmed through the annotation of the genomes of these bacteria. GU1.7.1 and GU3.1.1 are motile bacteria under the tested condition. All the isolates were able to produce acids from glucose and galactose without gas production, but none of them fermented xylose and lactose. GU1.7.1 and GU3.1.1 fermented maltose and saccharose. Only GU1.7.1 produced acid from sorbitol, and AFP5.1 from mannitol. This last bacterium was able to use thiosulfate as final electron acceptor as it was visualized through the production of H_2_S. The enzyme lysine decarboxylase is produced by the three isolates. Catalase was detected in GU1.7.1 and GU3.1.1. Meanwhile, only AFP5.1 was able to produce the enzyme ornithine decarboxylase, and a citrate permease that allow to this bacterium using citrate as sole carbon source. No indole production was detected for any bacteria.

**Table 2:** Polyphasic characterization of microorganisms isolated from samples from different places in Antarctica

Microorganism	GU1.7.1	GU3.1.1	AFP5.1
Genus	*Sphingomonas*	*Plantibacter*	*Pseudomonas*
Gram staining	−	+	−
Morphology	Rods	Rods	Rods
Motility test	+	+	−
Acid production from			
Glucose	+	+	+
Galactose	+	+	+
Xylose	−	−	−
Maltose	+	+	−
Lactose	−	−	−
Saccharose	+	+	−
Mannitol	−	−	+
Sorbitol	+	−	−
Gas production	−	−	−
H_2_S production	−	−	+
TSI agar	A/A	A/K	A/K
LIA agar	K/K	K/K	K/K
Lysine decarboxylase	+	+	+
Use of citrate	−	−	+
Indole production	−	−	−
Catalase	+	+	−
Ornithine decarboxylase	−	−	+

+, positive reaction; −, negative reaction; A, pH decrease; K, pH increase; ND, not determined

### Purification and molecular mass determination of AFPs

**Fig. 1:**
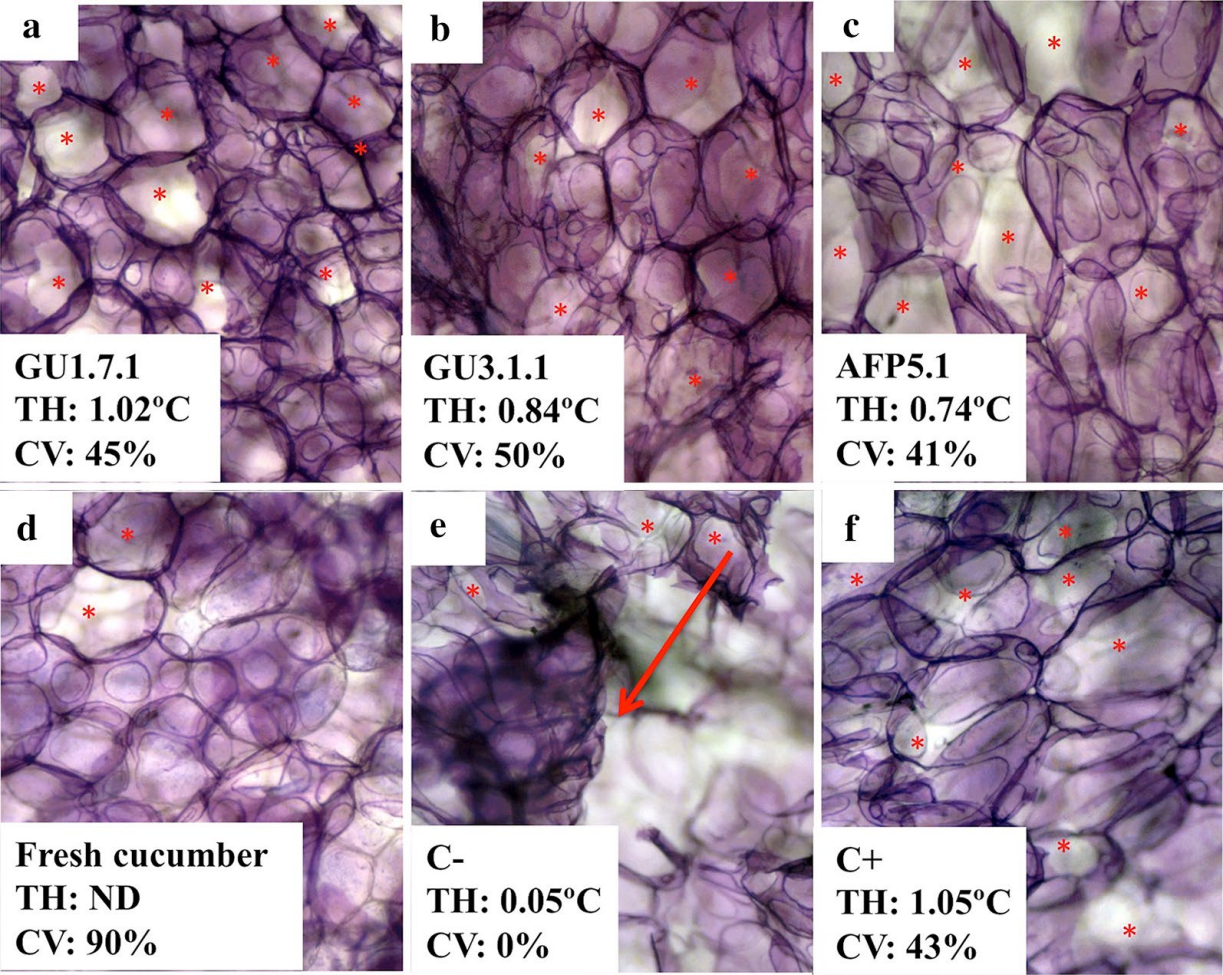
Effect of AFPs from GU1.7.1 (**a**), GU3.1.1 (**b**) and AFP5.1 (**c**) on cucumber. Negative (C−, **e**) and positive (C+, **f**) controls correspond to samples treated with buffer and a commercial AFP, respectively. Plant cells were stained with TBO. TH of AFPs from Antarctic isolates and plant cell viability (CV), determined by NR staining, are indicated in each image. A fresh cucumber sample (**d**) was included in order to compare results. *Red asterisks* represent small holes formed from small ice crystal; meanwhile, *red arrows* indicate high cellular damage product of cellular disruption due to the presence of big ice crystals. *ND* not determined

In this study, four AFPs from microorganisms GU1.7.1, GU3.1.1 and AFP5.1 were partially purified and their TH was measured (Fig. 1). After Q-Sepharose step, only one peak with TH was obtained from GU1.7.1 and AFP5.1 (these AFPs were named gu1A and afp5A, respectively), while two different fractions with TH were collected from GU3.1.1 crude extract (gu3A and gu3B). The apparent molecular mass was estimated by size exclusion chromatography using a Superdex 200 column. A significant loss of activity was measured after this step and no further purification steps were performed. Apparent molecular mass for gu1A, gu3B and afp5A were 50.9, 34.3 and 46.8 kDa, respectively. It was not possible to determine the molecular weight for gu3A due to the low quantity of protein obtained after Q-Sepharose step. These values are in agreement with the predicted molecular mass obtained from the aminoacid sequences, suggesting that these AFPs are monomers. Due to the low quantity of protein and reduced antifreeze activity of the fractions obtained from Superdex 200 column, further experiments were performed using active fractions obtained from Q-Sepharose column, which contain partially purified AFPs.

### Effect of AFPs on frozen zucchini and cucumber

**Fig. 2:**
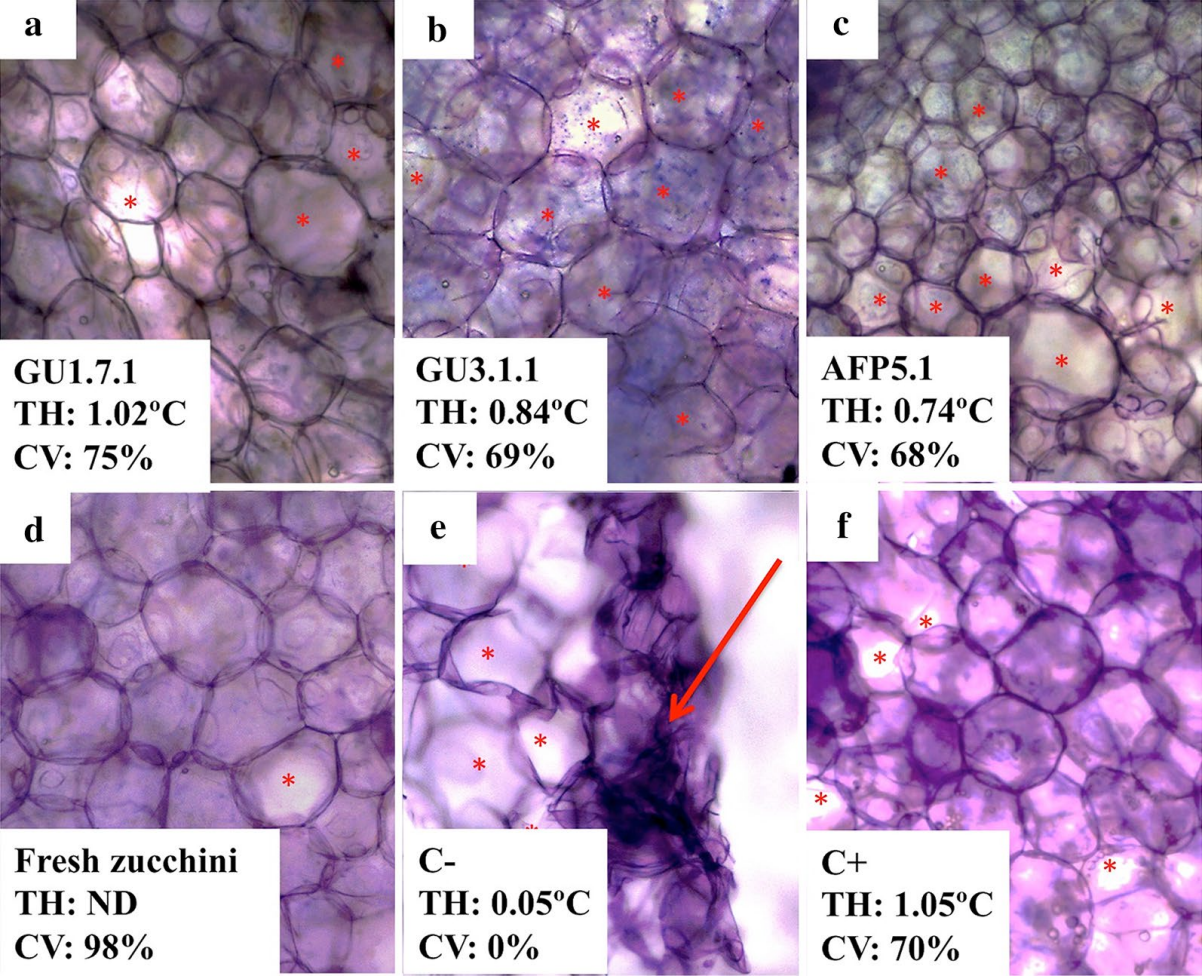
Effect of AFPs from GU1.7.1 (**a**), GU3.1.1 (**b**) and AFP5.1 (**c**) on zucchini. Negative (C−, **e**) and positive (C+, **f**) controls correspond to samples treated with buffer and a commercial AFP, respectively. Plant cells were stained with TBO. TH of AFPs from Antarctic isolates and plant cell viability (CV), determined by NR staining, are indicated in each image. A fresh zucchini sample (**d**) was included in order to compare results. *Red asterisks* represent small holes formed from small ice crystal; meanwhile, *red arrows* indicate high cellular damage product of cellular disruption due to the presence of big ice crystals. *ND* not determined

In order to determine the effect of these bacterial AFPs on frozen foods, small samples of zucchini and cucumber were cut and incubated in the presence of solutions containing different bacterial AFPs (Figs. 1a–c, 2a–c), a commercial AFP (Figs. 1f, 2f) or buffer at −20 °C (Figs. 1e, 2e). It was not possible to perform this experiment with gu3A due to the low quantity of protein obtained after Q-Sepharose step. After thawing, the samples were stained with TBO (to determine the damage on plant cell wall) or NR (to quantify the cell viability). In absence of any AFPs, the damage in cell walls of zucchini and cucumbers was significant, observing the loss of integrity of plant cells (red arrows, Figs. 1e, 2e). A protective effect was observed in samples treated with bacterial or commercial AFPs, as is evidence by the maintenance of cell walls in both fruits. Small holes are observed in some cells (red asterisk Figs. 1, 2) treated with the different AFPs, indicating cellular damage. These pores were barely visible in the cell walls when fresh cucumbers and zucchinis were stained with TBO (Figs. 1d, 2d).

Living cells were not observed when zucchini and cucumber samples were treated with buffer solution. On the other hand, living cells were detected when zucchini and cucumber were treated with bacterial or commercial AFPs. A less percentage of living cells were counted in cucumber samples in comparison to zucchini.

### Antifreeze sequences identification

**Fig. 3:**
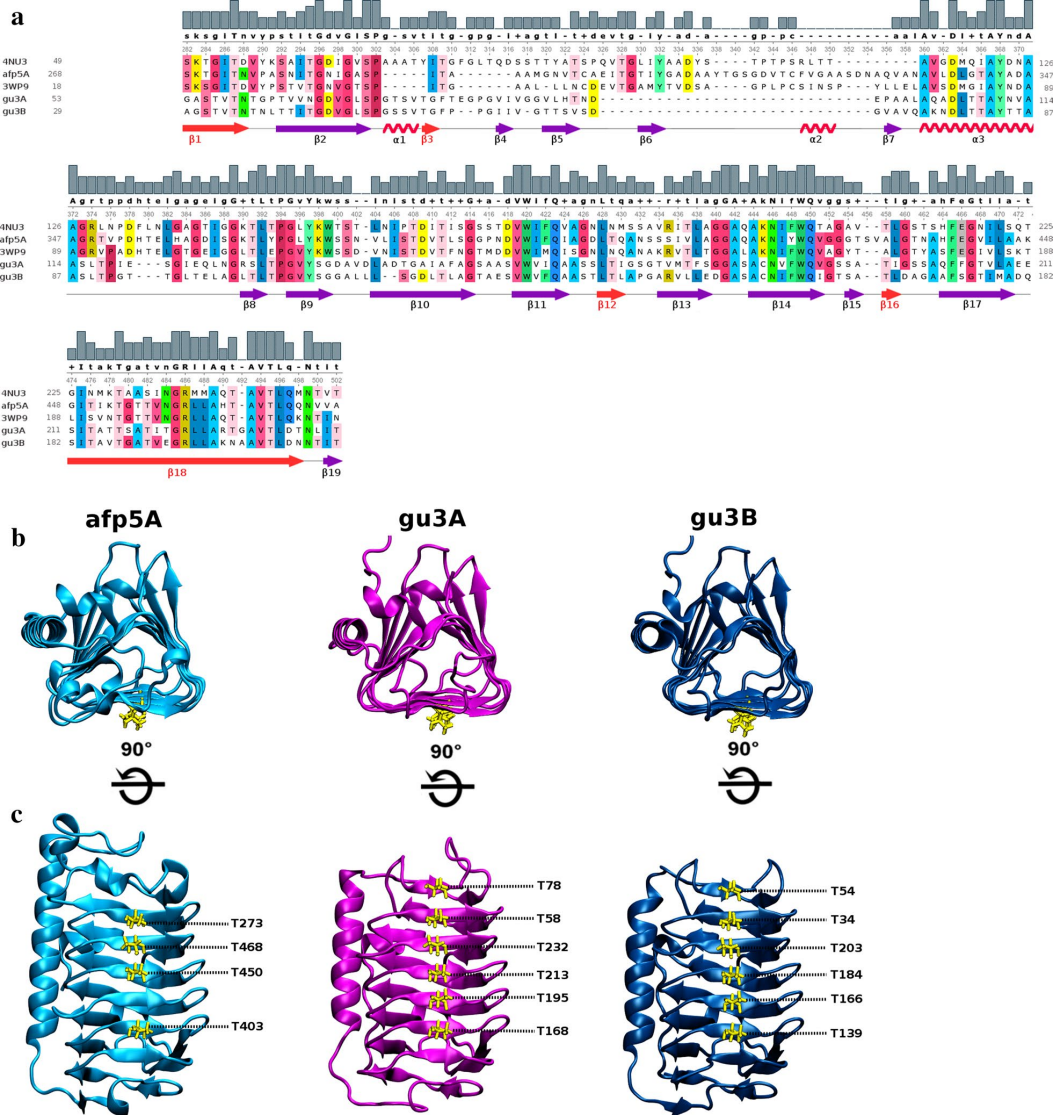
Cartoon representations of AFP models of afp5A (*cyan*), gu3A (*magenta*), and gu3B (*blue*). **a** Front view of triangular prisms. **b** Stereoviews showing the putative binding surface with ordered threonine residues (*yellow*). Images were generated using VMD. **c** Multiple sequence alignment of class III AFPs of the putative antifreeze proteins gu3A and gu3B (GU3.1.1) and afp5A (AFP5.1) and their respective templates for homology modeling, 3WP9 (hyperactive antifreeze protein from *Colwellia* sp.) and 4NU3, (hyperactive antifreeze protein from *Flavobacterium frigoris*). The region shown in the alignment corresponds only to the DUF3494 conserved domains of the sequences. Conserved amino acids in at least three of the aligned sequences are shown in *colors*, being each amino acid colored with a *different color*. *Histogram bars and uppercase/lowercase letters* in the consensus sequence indicate the level of conservation of the residues at each position of the alignment. *Arrows and ribbons* represent β-strands and α-helices, respectively. β-strands in *red* (β1, β3, β12, β16, β18) correspond to the β-strands located on the putative ice-binding surface of the AFPs

BLASTp search against a local database of antifreeze proteins and realated proteins (see methods) allowed identifying four putative AFP-coding genes. Two different sequences denominated gu3A (413 amino acids) and gu3B (360 amino acids) found in GU3.1.1 genome did match to the same sequence in the database (GenBank accession ALG05172.1), which corresponds to a protein annotated as an ice-binding protein from an unidentified bacterium (not published), with 53 and 63% identity, respectively. Meanwhile, only one sequence denominated afp5A (479 amino acids) could be identified as an ice-binding protein in AFP5.1 genome, nevertheless, these sequence matched to a well studied AFP from the Antarctic sea ice bacterium *Colwellia* sp. SLW05 [12] with 55% identity. Finally, the search in GU1.7.1 genome revealed a unique match sequence with low identity (37%) to an antifreeze-protein from the Antarctic bacterium *Marinomonas primoryensis* [10]. Multiple sequence alignment between these three sequences revealed 49 conserved residues (Additional file 1: Figure S1), which decreases to 37 when amino acid sequences of 4NU3 (Uniprot entry: H7FWB6) and 3WP9 (Uniprot entry: A5XB26) are included in the alignment (Fig. 3a). Motifs of regularly spaced-threonines T-x-x-T and T-x-T are present in the predicted β-strands (Fig. 3a).

### Homology modeling

The sequence analysis of putative AFPs from GU3.1.1 and AFP5.1 using NCBI’s conserved Domains Database (CDD) revealed that they share a large conserved domain of unknown function (DUF3494), which is common to the type 1 ice-binding proteins [9], at positions 268–474 for afp5A, 53–239 for gu3A and 29–210 for gu3B. For the homology modeling of these three sequences, only DUF3493 domains were considered, because this was the only region with known structural information covered by the alignments performed by the template search tools in the SWISSMODEL server.

3D models of gu3A and gu3B were generated using as template the crystal structure of a hyperactive ice binding protein from the Antarctic bacterium *Flavobacterium frigoris* PS1 (PDB Id: 4NU3), having GMQE scores of 0.75 (34% sequence identity, 99% coverage) and 0.76 (35% sequence identity, 100% coverage), respectively. 3D model of afp5A was generated using the crystal of an antifreeze protein from the Antarctic sea bacterium *Colwellia* sp. (PDB Id: 3WP9), having a GMQE score of 0.77 (56% sequence identity, coverage 96%). The oligomeric state inferred from the templates for the three models is monomeric. Ramachandran plots for the generated models revealed that majority of amino acids (>90%) are positioned in favorable regions for the three structures (Additional file 2: Figure S2). The observed shape of these three AFPs is a triangular prism composed of β-sheets (Fig. 3b). The putative ice-binding surface is composed of six parallel β-strands in afp5A and seven in gu3A and gu3B as shown in Fig. 3c.

### Docking and molecular dynamics simulations

**Fig. 4:**
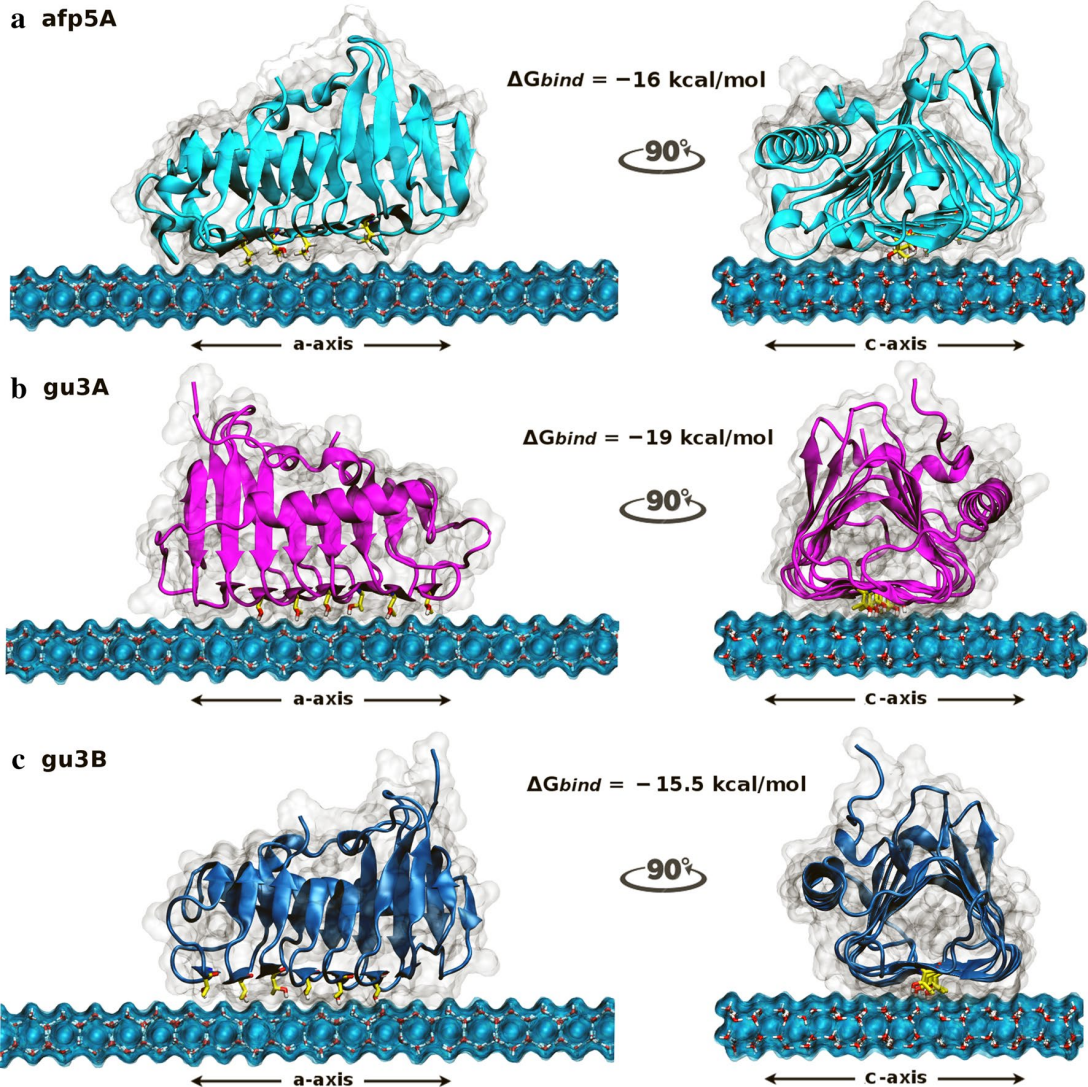
Side views of the final docking orientations of afp5A (**a**), gu3A (**b**) y gu3B (**c**). a-Axis view (*left*) and c-axis (*right*) of the docked conformations of AFPs over the primary prism plane of ice. Docking binding energies obtained are shown for each protein. Regularly spaced threonine residues in the ice-binding surface of the proteins are shown in *yellow*. All docking simulations were performed with Autodock Vina. All images were generated with VMD

A molecular docking/molecular dynamics approach was used in order to obtain insights into the molecular interactions between the structural models of AFPs generated and ice at the interface ice/water. AFPs were positioned over the primary prism plane of a freely available ice layer model [39] inside a 44 × 62 × 47 Å grid box where rigid docking was performed. The resulting highest binding free energies from the conformational search and affinity scoring were −16, −19 and −15.5 kcal/mol for afp5A, gu3A, and gu3B, respectively. The three docked models showed preference for the putative ice-binding surface with ordered threonine residues contacting the ice plane (Fig. 4) and the three of them adopted a similar orientation over the ice layer (Additional file 3: Figure S3).

**Fig. 5:**
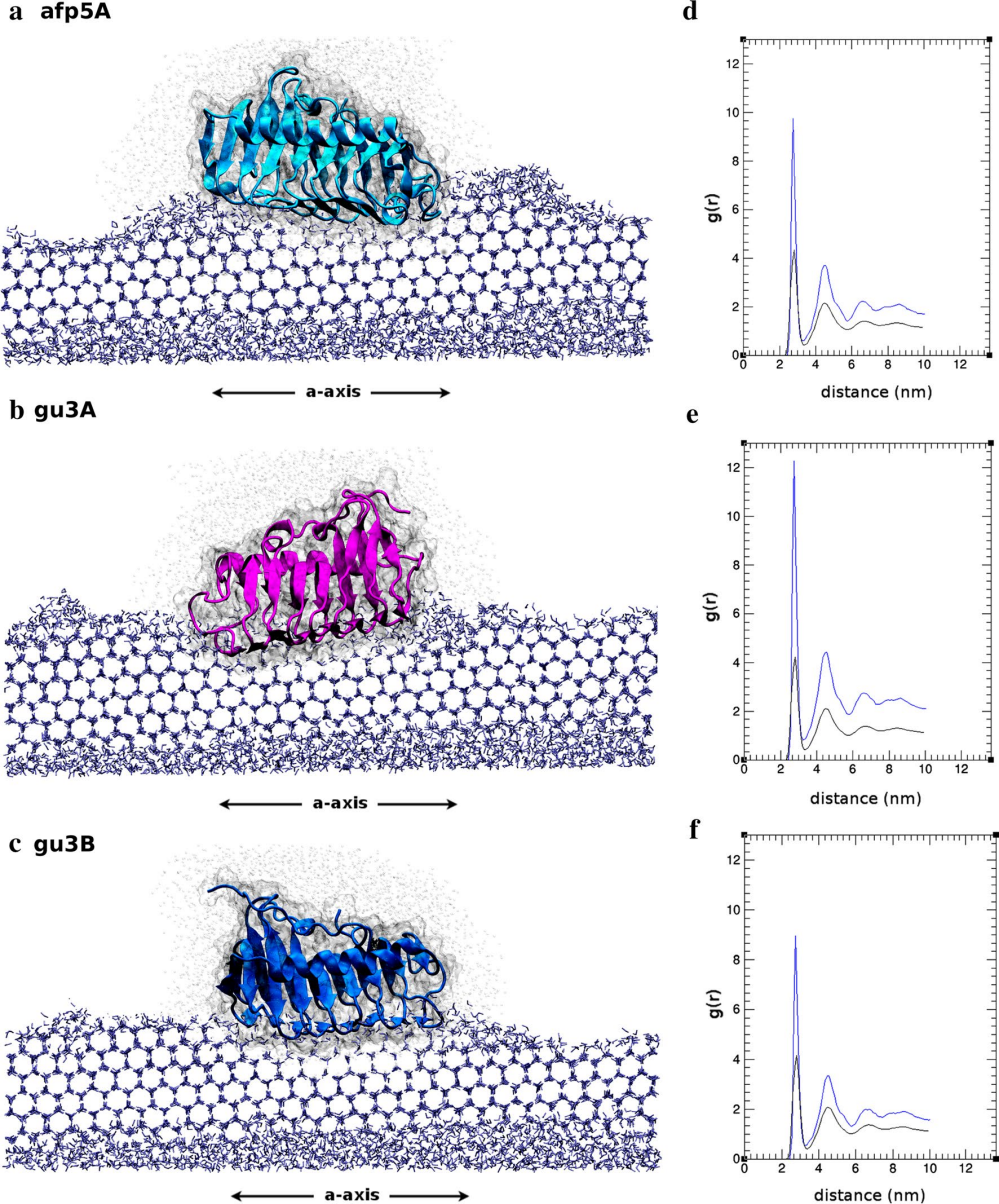
Front view (a-axis) of simulated AFPs afp5A (**a**), gu3A (**b**), gu3B (**c**) and the corresponding oxygen–oxygen radial distribution functions (**d**–**f**). In RDF graphs *blue lines* correspond to g(r) distribution calculated at the end (150 ns) of each simulation whilst the *black lines* correspond to the same function calculated at the start of molecular dynamics simulations. Simulations were carried out using NAMD software. All images were generated using VMD

Besides the orientation and positioning of proteins over the ice layer, no further inferences concerning antifreeze behavior could be made from docking results. Coordinates obtained were used as starting point for molecular dynamics simulations over growing ice lattices. Calculations were performed according to protocol described in methods for the three systems: gu3A-ice; gu3B-ice; afp5A-ice. After 150 ns dynamics simulations, it was possible to observe the ice growth in each system but no significant difference could be observed with the naked eye. Thereby, as a way to describe the structural arrangement of ice-like water molecules in the solvation shells surrounding the proteins, radial distribution functions g(r) were used. In particular, g_OO_(r), which corresponds to the radial distribution as a function of the distance between two oxygen atoms from water molecules, was used as a measure of the ice formation. It has been well reported that radial distribution function for crystalline ice is markedly different from that of liquid water [47] and has been previously used to characterize the antifreeze ability of a thermal hysteresis protein [48]. We analyzed the behavior of water molecules located at 15 Å from every atom of the proteins. Thus, what g_OO_(r) plot is representing is the probability of finding another oxygen atom at a given radial distance from the first, but considering only water molecules within a distance of 15 Å of the protein. The RDF patterns observed in Fig. 5d, e, f are in very good agreement with those reported by Soper [47] for ice. The intensity of each peak indicates the abundance of ice-like water molecules in each solvation shell. Thus, at the end of each simulation, the intensity of the peaks for the three RDFs presented is higher than it was at the starting point, indicating ice-like water organization of molecules surrounding the proteins that were different at the start of dynamics. g_OO_(r) analysis considering the solvation shell of 15 Å shed light on a possibly more efficient antifreezing behavior of gu3B. RDF for this protein shows a lower peak near 4 and 5 nm compared to gu3A and afp5A, revealing a lower of ice-like water molecules around this protein. In contrast, radial distribution peaks from afp5A and gu3A are both higher compared to those of gu3B. Captures of the final frames obtained with molecular dynamics are shown in Fig. 5.

## Discussion

Psychrophilic organisms produce AFPs in order to prevent ice growth during freezing. Some studies have demonstrated that these proteins are extremely effective in the inhibition of recrystallization of ice even at very low concentrations [49]. However, their working mechanism is not well understood. Moreover, their properties offer the opportunity to exploit AFPs for biotechnological applications, especially in food industry. In frozen food products, freezing provides a multitude of small ice crystals, but it is also important to prevent the formations of large ice crystals [50]. AFPs from Antarctic bacteria GU1.7.1, GU3.1.1 and AFP5.1 were able to reduce the damage associated to ice, probably through the reduction of the size of large ice crystals in buffer solution. This phenomenon was evidenced when zucchini and cucumbers tissues were treated with a commercial and bacterial AFPs, where small pore were (red asterisk Figs. 1, 2) formed on plant cell walls of treated tissues in comparison to the important damage observed in absent of any AFPs. In this sense, the use of AFPs may offer the opportunity for the storage of different fruits in frozen conditions. However, the external addition of AFPs could only protect the most superficial tissues, and for this reason it is necessary to introduced AFPs-coding genes in plant organisms in order to produce fruits resistant to freezing–thawing process. Moreover, the genetically modified plants with AFPs-coding genes may improve the texture and quality of frozen foods [20].

AFPs have been isolated and characterized from different sources, including fish, insects, plants among others, and correspond to a structurally diverse group of proteins. These proteins are different in their structures, from amino acid sequences to tertiary structure, and they all bind to ice crystals in order to reduce the freezing point of water [51], but some of them have the ability to help organisms tolerate freezing through the inhibition of ice recrystallization, or to allow adhering their host to ice. This has led to the use of the more inclusive descriptive term of Ice Binding Proteins [52]. Protein modeling of afp5A, gu3A and gu3B revealed a high structural similarity to β-helix AFPs (Fig. 3) consisting of one α-helix and β-sheets arranged in a triangular prism form. The putative ice binding sites is composed of six β-sheets in afp5A and seven for AFPs of GU3.1.1 isolate. Similar structures have been described for AFP of *Tenebrio molitor* [53] and *Choristoneura fumiferana* [17]. These last proteins possess a β-helix with a triangular cross-section and rectangular sides, containing threonine residues arranged in the ice-binding side of the prism in regular array of TXT motifs. Models for gu3A, gu3B and afp5A show threonine residues ordered in a linear disposition in one side of the prism (Fig. 3c) similar to AFPs from *T. molitor* and *C. fumiferana*. Moreover, the majority of these residues are arranged in TXT motif, but some of them are distributed in almost perfect TXT motifs, where threonine residues are spaced for two amino acids. These motifs have been implicated in ice binding; due to their replacement for leucine strongly decrease the antifreeze activity [17]. Ice–AFP interaction requires that threonine residues were distinctly positioned in the ice-binding side as in the case of afp5A, gu3A and gu3B. Hydroxyl groups from threonine protrude from the center of the AFP and are exposed and accessible to interact with the ice crystal [54]. AFPs studied in this work (Figs. 4, 5) clearly show complementarity between ice prism planes and regularly spaced threonine residues along the ice binding surfaces of each protein.

Molecular dynamics simulations of AFPs allowed establishing interaction of Antarctic AFPs from isolates GU3.1.1 and AFP5.1 at the water/ice interface (Fig. 5). Threonine residues seems to be important for this interaction and when radial distribution function of oxygen was calculated and analyzed after 150 ns of molecular simulation, it revealed distinctive antifreeze behaviors among them, suggesting a more efficient antifreeze activity for gu3B, due to the radial distribution peaks for ice in presence of this AFP was reduced in comparison to gu3A and afp5A (Fig. 5d–f, blue line), indicating a smaller quantity of water molecules in solid state. However, the best binding affinity was observed for gu3A (−19 kcal/mol). In contrast to gu3A and gu3B, afp5A lacks two threonine residues in β3 and β16 that are present in the former and might have influence in the affinity for ice, as can be seen in Fig. 5a where this AFP is irregularly bounded to the ice lattice. However, binding affinity of afp5 is similar to that of gu3B and shows a more regular binding.

In conclusion, AFPs from the Antarctic microorganisms GU1.7.1, GU3.1.1 and AFP5.1 protect cellular structures of zucchini and cucumber showing their potential in frozen foods.

Models of gu3A, gu3B and afp5A indicate that these AFPs possess a similar structure to β-helix AFPs, and in silico analyzes suggest that gu3B is the most efficient AFPs in order to avoid the formation of big ice crystals, even when gu3A showed a higher affinity for ice. By determining the interaction of these proteins at the ice/water interface, it will make possible to understand the process of adaptation of psychrophilic bacteria to ice in Antarctica.

## Additional files

**Additional file 1: Figure S1.** Multiple sequence alignment between the DUF3494 domains of the identified antifreeze proteins gu3A, gu3B and afp5A. Asterisks (*) indicate positions with fully conserved residues; colons (:) indicate conservation of residues with strong similar properties; periods (.) indicate conservation of weakly similar residues. Alignment was performed using ClustalW.

**Additional file 2: Figure S2.** Ramachandran plot analysis of the dihedral angles PSI (ψ) and PHI (ɸ) of the generated models for (a) afp5A, (b) gu3A and (c) gu3B obtained by RAMPAGE. The three plots show favorable positioning of amino acids for the different models generated.

**Additional file 3: Figure S3.** Radial distribution functions of a) water at 298 K (liquid water) and b) 220 K (ice) determined experimentally from X-ray and neutron diffraction by Soper [47]. Images were obtained and adapted from http://rkt.chem.ox.ac.uk/lectures/liqsolns/liquids.html.

## Abbreviations

AFP: antifreeze proteins
TH: thermal hysteresis
TBO: toluidine blue O
NR: neutral red
CV: cell viability

## References

[1] Wilkins D, Yau S, Williams TJ, Allen MA, Brown MV, DeMaere MZ, Lauro FM, Cavicchioli R (2013). Key microbial drivers in Antarctic aquatic environments. FEMS Microbiol Rev.

[2] D’Amico S, Collins T, Marx JC, Feller G, Gerday C (2006). Psychrophilic microorganisms: challenges for life. EMBO Rep.

[3] De Maayer P, Anderson D, Cary C, Cowan DA (2014). Some like it cold: understanding the survival strategies of psychrophiles. EMBO Rep.

[4] Devries A, Wohlschl D (1969). Freezing resistance in some Antarctic fishes. Science.

[5] Duman J, Devries A (1974). Freezing resistance in winter flounder *Pseudopleuronectes americanus*. Nature.

[6] Raymond J, Devries A (1977). Adsorption inhibition as a mechanism of freezing resistance in polar fishes. PNAS.

[7] Hoshino T, Kiriaki M, Ohgiya S, Fujiwara M, Kondo H, Nishimiya Y, Yumoto I, Tsuda S (2003). Antifreeze proteins from snow mold fungi. Can J Bot.

[8] Janech M, Krell A, Mock T, Kang J, Raymond JA (2006). Ice-binding proteins from sea ice diatoms (Bacillariophyceae). J Phycol.

[9] Raymond J (2014). The ice-binding proteins of a snow alga, *Chloromonas brevispina*: probable acquisition by horizontal gene transfer. Extremophiles.

[10] Garnham C, Gilbert J, Hartman C, Campbell R, Laybourn-Parry J, Davies P (2008). A Ca^2+^-dependent bacterial antifreeze protein domain has a novel beta-helical ice-binding fold. Biochem J.

[11] Gilbert J, Hill P, Dodd C, Laybourn-Parry J (2004). Demonstration of antifreeze protein activity in Antarctic lake bacteria. Microbiology.

[12] Raymond J, Fritsen C, Shen K (2007). An ice-binding protein from an Antarctic sea ice bacterium. FEMS Microbiol Ecol.

[13] Wilson S, Kelley D, Walker V (2006). Ice-active characteristics of soil bacteria selected by ice-affinity. Environ Microbiol.

[14] Celik Y, Graham LA, Mok YF, Bar M, Davies PL, Braslavsky I (2010). Superheating of ice crystals in antifreeze protein solutions. PNAS.

[15] Kristiansen E, Zachariassen KE (2005). The mechanism by which fish antifreeze proteins cause thermal hysteresis. Cryobiology.

[16] DeVries A (1971). Glycoproteins as biological antifreeze agents in Antarctic fishes. Science.

[17] Graether SP, Kuiper MJ, Gagné SM, Walker VK, Jia Z, Sykes BD, Davies PL (2000). β-Helix structure and ice-binding properties of a hyperactive antifreeze protein from an insect. Nature.

[18] Christner BC (2010). Bioprospecting for microbial products that affect ice crystal formation and growth. Appl Microbiol Biotechnol.

[19] Jeon SM, Naing AH, Park KI, Kim CK (2015). The effect of antifreeze protein on the cryopreservation of *chrysanthemums*. Plant Cell Tiss Organ Cult.

[20] Zhou H, Infante-Ferreira CA. The effect of type-III antifreeze proteins (AFPs) on CO_2_ hydrate slurry formation. International Refrigeration and Air Conditioning Conference. 2014. http://docs.lib.purdue.edu/iracc/1473/. Accessed 22 Dec 2014.

[21] Griffith M, Ewart K (1995). Antifreeze proteins and their potential use in frozen foods. Biotechnol Adv.

[22] Wilson S, Grogan P, Walker V (2012). Prospecting for ice association: characterization of freeze–thaw selected enrichment cultures from latitudinally distant soils. Can J Microbiol.

[23] McCord JM, Keele BB, Fridovich I (1971). An enzyme based theory of obligate anaerobiosis: the physiological role of superoxide dismutase. PNAS.

[24] Frank JA, Reich CI, Sharma S, Weisbaum JS, Wilson BA, Olsen GJ (2008). Critical evaluation of two primers commonly used for amplification of bacterial 16S rRNA genes. Appl Environ Microbiol.

[25] Correa-Llantén D, Amenábar M, Muñoz P, Monsalves M, Castro M, Blamey J (2014). *Alicyclobacillus* sp. strain CC2, a thermo-acidophilic bacterium isolated from Deception Island (Antarctica) with important superoxide dismutase activity. APS.

[26] Braslavsky I, Drori R (2013). LabVIEW-operated novel Nanoliter Osmometer for ice binding protein investigations. J Vis Exp.

[27] Bradford M (1976). Rapid and sensitive method for the quantitation of microgram quantities of protein utilizing the principle of protein. Anal Biochem.

[28] Gonzalez ME, Jernstedt JA, Slaughter DC, Barrer DM (2010). Microscopic quantification of cell integrity in raw and processed onion parenchyma cells. J Food Sci.

[29] Zerbino DR (2010). Using the velvet *de novo* assembler for short-read sequencing technologies. Curr Protoc Bioinform.

[30] Aziz RK, Bartels D, Best AA, DeJongh M, Disz T, Edwards RA, Formsma K, Gerdes S, Glass EM, Kubal M, Meyer F, Olsen GJ, Olson R, Osterman AL, Overbeek RA, McNeil LK, Paarmann D, Paczian T, Parrello B, Pusch GD, Reich C, Stevens R, Vassieva O, Vonstein V, Wilke A, Zagnitko O (2008). The RAST server: rapid annotation using subsystems technology. BMC Genom.

[31] Hyatt D, Chen GL, Locascio PF, Land ML, Larimer FW, Hauser LJ (2010). Prodigal: prokaryotic gene recognition and translation initiation site identification. BMC Bioinform.

[32] Altschul SF, Gish W, Miller W, Myers EW, Lipman DJ (1990). Basic local alignment search tool. J Mol Biol.

[33] Marchler-Bauer A, Derbyshire MK, Gonzales NR, Lu S, Chitsaz F, Geer LY, Geer RC, He J, Gwadz M, Hurwitz DI, Lanczycki CJ, Lu F, Marchler GH, Song JS, Thanki N, Wang Z, Yamashita RA, Zhang D, Zheng C, Bryant SH (2015). CDD: NCBI’s conserved domain database. Nucleic Acids Res.

[34] Notredame C, Higgins DG, Heringa J (2000). T-Coffee: a novel method for multiple sequence alignments. J Mol Biol.

[35] Pei J, Kim BH, Grishin NV (2008). PROMALS3D: a tool for multiple sequence and structure alignment. Nucleic Acids Res.

[36] Biasini M, Bienert S, Waterhouse A, Arnold K, Studer G, Schmidt T, Kiefer F, Gallo Cassarino T, Bertoni M, Bordoli L, Schwede T (2014). SWISS-MODEL: modelling protein tertiary and quaternary structure using evolutionary information. Nucleic Acids Res.

[37] Phillips JC, Braun R, Wang W, Gumbart J, Tajkhorshid E, Villa E, Chipot C, Skeel RD, Kalé L, Schulten K (2005). Scalable molecular dynamics with NAMD. J Comput Chem.

[38] Lovell SC, Davis IW, Arendall WB, de Bakker PIW, Word JM, Prisant MG, Richardson JS, Richardson DC (2003). Structure validation by Cα geometry: ϕ, ψ and Cβ deviation. Proteins.

[39] Kuiper MJ, Morton CJ, Abraham SE, Gray-Weale A (2015). The biological function of an insect antifreeze protein simulated by molecular dynamics. Elife.

[40] Morris GM, Ruth H, Lindstrom W, Sanner MF, Belew RK, Goodsell DS, Olson AJ (2009). Software news and updates AutoDock4 and AutoDockTools4: automated docking with selective receptor flexibility. J Comput Chem.

[41] Trott O, Olson AJ (2010). AutoDock Vina: improving the speed and accuracy of docking with a new scoring function, efficient optimization and multithreading. J Comput Chem.

[42] MacKerel AD, Brooks CL, Nilsson L, Roux B, Won Y, Karplus M (1998). CHARMM: the energy function and its parameterization with an overview of the program. Encycl Comput Chem.

[43] Martyna G, Tobias D, Klein M (1994). Constant pressure molecular dynamics algorithms. J Chem Phys.

[44] Miyamoto S, Kollman PA (1992). Settle: an analytical version of the SHAKE and RATTLE algorithm for rigid water models. J Comput Chem.

[45] Andersen HC (1983). Rattle: a “velocity” version of the shake algorithm for molecular dynamics calculations. J Comput Phys.

[46] Darden T, York D, Pedersen L (1993). Particle mesh Ewald: an N·log(N) method for Ewald sums in large systems. J Chem Phys.

[47] Soper AK (2000). The radial distribution functions of water and ice from 220 to 673 K and at pressures up to 400 MPa. Chem Phys.

[48] Yang C, Sharp KA (2004). The mechanism of the type III antifreeze protein action: a computational study. Biophys Chem.

[49] Zalis S, Bar Dolev M, Braslavsky I (2013). Inhibition of ice recrystallization by antifreeze proteins. Cryobiology.

[50] Zachariassen KE, Lundheim R, Margesin R, Schinner F (1998). Applications of antifreeze proteins. Biotechnological applications of cold-adapted organisms.

[51] Davies PL, Baardsnes J, Kuiper MJ, Walker VK (2002). Structure and function of antifreeze proteins. Philos Trans R Soc Lond B.

[52] Bar Dolev M, Braslavsky I, Davies PL (2016). Ice-binding proteins and their function. Annu Rev Biochem.

[53] Daley ME, Spyracopoulos L, Jia Z, Davies PL, Sykes BD (2002). Structure and dynamics of a β-helical antifreeze protein. Biochemistry.

[54] Leinala EK, Davies PL, Jia Z (2002). Crystal structure of β-helical antifreeze protein points to a general ice binding model. Structure.

