# Supplementary figures and images for: Structure and application of antifreeze proteins from Antarctic bacteria
Source: Microb Cell Fact. 2017 Aug 7;16:138. doi: 10.1186/s12934-017-0737-2 (PMC5547475; doi:10.1186/s12934-017-0737-2)

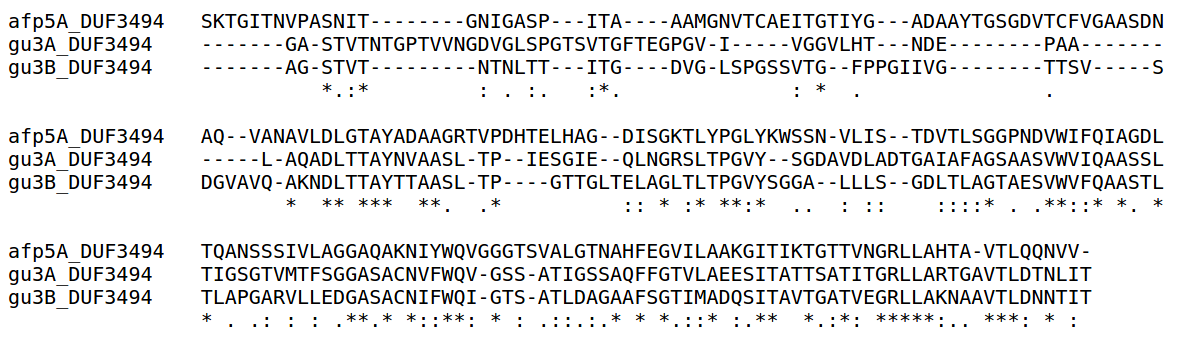

Supplement: Supplementary file 1 — Additional file 1: Figure S1. Multiple sequence alignment between the DUF3494 domains of the identified antifreeze proteins gu3A, gu3B and afp5A. Asterisks (*) indicate positions with fully conserved residues; colons (:) indicate conservation of residues with strong similar properties; periods (.) indicate conservation of weakly similar residues. Alignment was performed using ClustalW. [file 12934_2017_737_MOESM1_ESM.png]

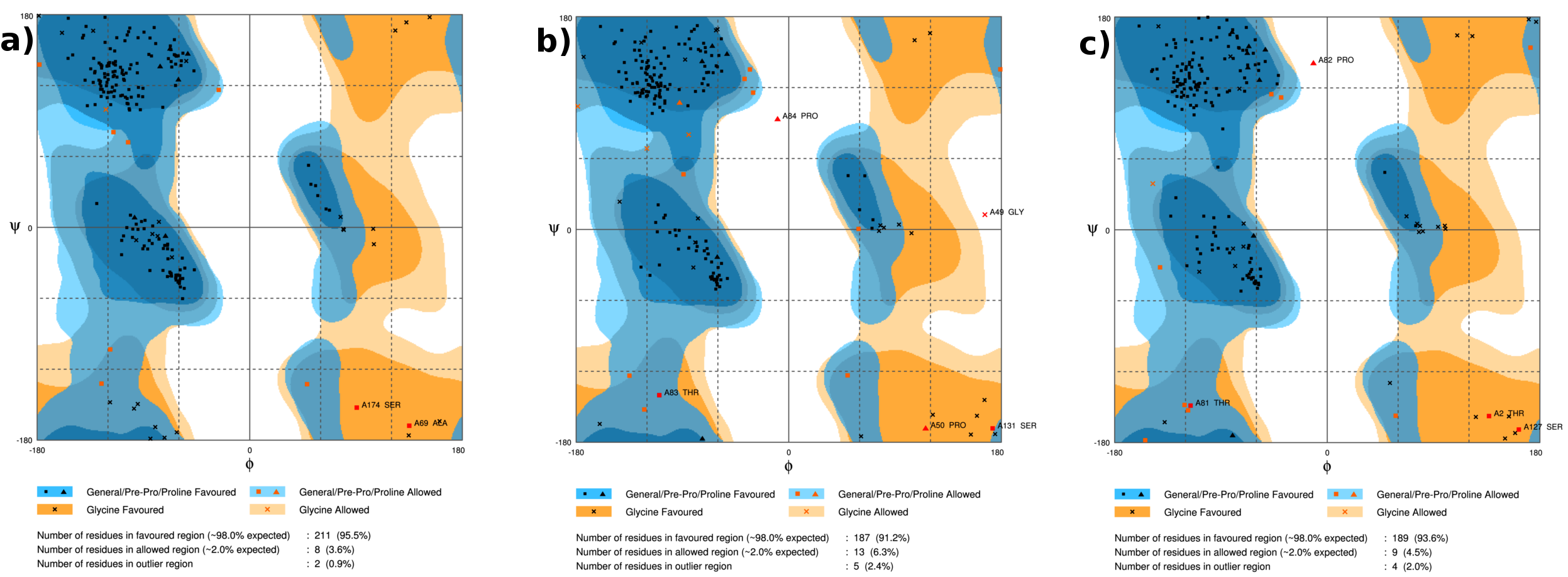

Supplement: Supplementary file 2 — Additional file 2: Figure S2. Ramachandran plot analysis of the dihedral angles PSI (ψ) and PHI (ɸ) of the generated models for (a) afp5A, (b) gu3A and (c) gu3B obtained by RAMPAGE. The three plots show favorable positioning of amino acids for the different models generated. [file 12934_2017_737_MOESM2_ESM.png]

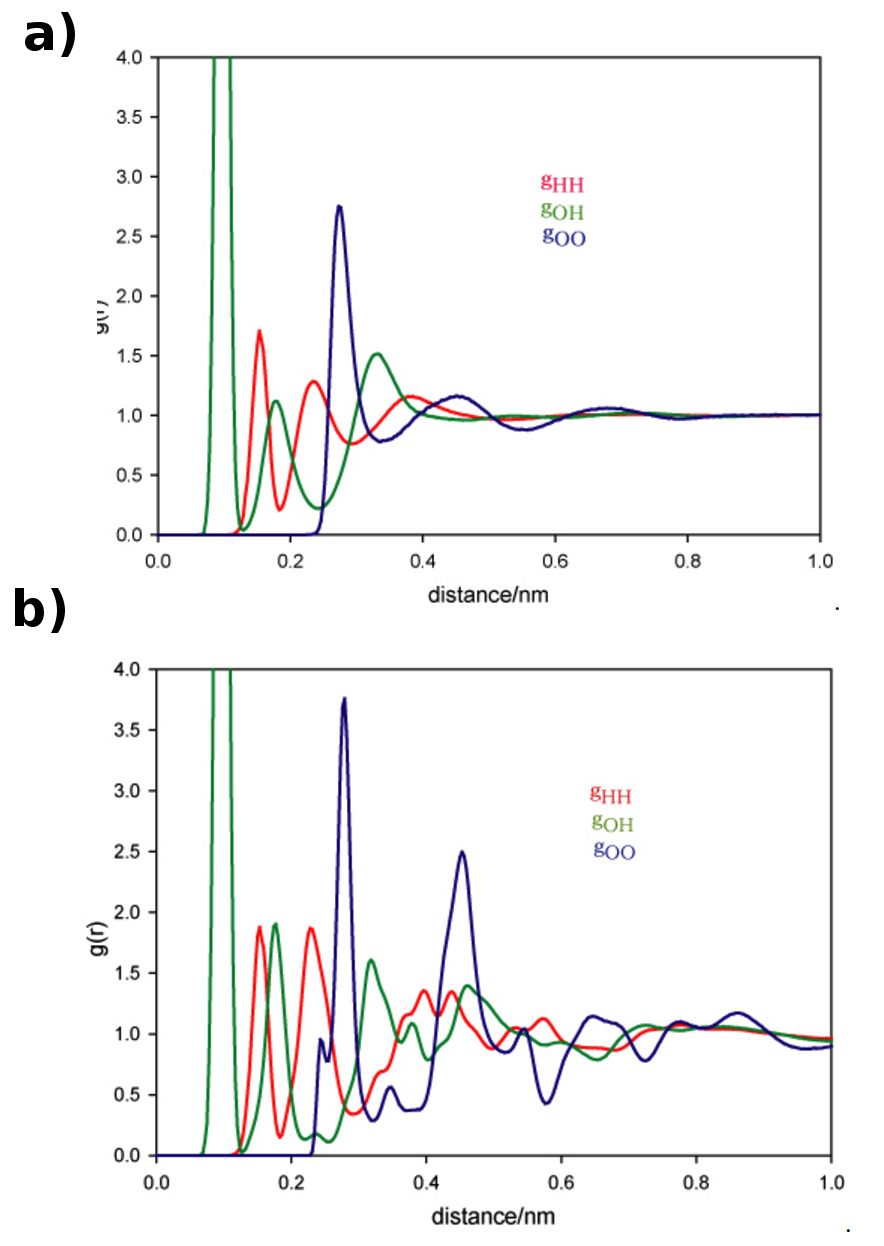

Supplement: Supplementary file 3 — Additional file 3: Figure S3. Radial distribution functions of a) water at 298 K (liquid water) and b) 220 K (ice) determined experimentally from X-ray and neutron diffraction by Soper [47]. Images were obtained and adapted from http://rkt.chem.ox.ac.uk/lectures/liqsolns/liquids.html. [file 12934_2017_737_MOESM3_ESM.png]
